# Gluten-Free Breadsticks Fortified with Phenolic-Rich Extracts from Olive Leaves and Olive Mill Wastewater

**DOI:** 10.3390/foods10050923

**Published:** 2021-04-22

**Authors:** Paola Conte, Simone Pulina, Alessandra Del Caro, Costantino Fadda, Pietro Paolo Urgeghe, Alessandra De Bruno, Graziana Difonzo, Francesco Caponio, Rosa Romeo, Antonio Piga

**Affiliations:** 1 Department of Agricultural Sciences, Università degli Studi di Sassari, Viale Italia 39/A, 07100 Sassari, Italy; spulina1@uniss.it (S.P.); delcaro@uniss.it (A.D.C.); cfadda@uniss.it (C.F.); paolou@uniss.it (P.P.U.); pigaa@uniss.it (A.P.); 2 Department of Agraria, University Mediterranea of Reggio Calabria, 89124 Reggio Calabria, Italy; alessandra.debruno@unirc.it (A.D.B.); rosa.romeo@unirc.it (R.R.); 3 Department of Soil Plant and Food Sciences, University of Bari Aldo Moro, Via Amendola 165/A, 70126 Bari, Italy; graziana.difonzo@uniba.it (G.D.); francesco.caponio@uniba.it (F.C.)

**Keywords:** breadsticks, gluten-free, olive oil by-products, antioxidants, oxidation stability

## Abstract

Nowadays, food processing by-products, which have long raised serious environmental concerns, are recognized to be a cheap source of valuable compounds. In the present study, incorporation of phenolic-rich extracts (500 and 1000 mg kg^−1^) from olive leaves (OL) and olive mill wastewater (OMW) into conventional gluten-free formulations has been exploited as a potential strategy for developing nutritious and healthy breadsticks with extended shelf-life. To this end, moisture, water activity (a_w_), visual and textural properties, the composition of biologically active compounds (soluble, insoluble, and bio-accessible polyphenols), antioxidant activity, oxidation stability, and consumer preference of the resulting breadsticks were investigated. Fortified breadsticks had higher moisture and a_w_, lower hardness, and similar color in comparison to the control, especially in the case of OL extract supplementation. All enriched formulations significantly affected the phenolic composition, as evidenced by the decrease in insoluble/soluble polyphenols ratio (from 7 in the control up to 3.1 and 4.5 in OL and OMW, respectively), and a concomitant increase in polyphenol bio-accessibility (OL: 14.5–23% and OMW: 10.4–15% rise) and antioxidant activity (OL: 20–36% and OMW: 11–16% rise). Moreover, a significant shelf-life extension was observed in all fortified breadsticks (especially in case of OMW supplementation). Sensory evaluation evidenced that 61% of the assessors showed a marked, but not significant, tendency to consider the sample supplemented with high levels of OL as a more palatable choice.

## 1. Introduction

For a long time, food processing waste and by-products have been of great concern for both agro-food industries and society due to the environmental and economic impacts induced by their safe disposal [1]. In the past few year, however, this view has changed in order to recognize such industry by-products as a potential and cheap source of both nutraceuticals and functional compounds (mainly bio-phenols) to be re-used in the development of highly-nutritious and healthy foods with extended shelf-life [2]. In addition, re-using products that are wasted during industrial processing and transformation could reduce food waste level, contribute to environmental preservation, and provide long-term sustainability to the food production systems [3].

The olive oil industry, apart from its main valuable product, usually produces–in a short period of time (from November to February)–huge amounts of other waste, such as olive leaves (OL), thin branches, woods, olive mill wastewater (OMW) and olive pomace, whose management, treatment and disposal are often the cause of major problems for olive oil producers [4,5]. The first residue generated during the virgin olive oil extraction process is represented by the OL, which are accumulated in large quantities (about 10% of the total weight of the harvested olives) during the early stages of olive cleaning or during pruning of olive trees [6]. These lignocellulosic residues are a rich source of phenolic compounds (at about 2.5%), mainly oleuropein, whose content can exceed 15% in dry material, but also verbascoside, hydroxy-tyrosol, tyrosol, caffeic acid, vanillic acid, luteolin, rutin, and apigenin, which are well known for their recognized antioxidant and antimicrobial activity [7,8,9]. OL also contain water (51%), carbohydrates (27%), protein (7%), crude fiber (7%), oil (3%), and ashes (2.5%) [10].

However, the most polluting by-product produced by the olive oil extraction industry is represented by the liquid effluent OMW, which is generated during the separation phase of the oil from the malaxed paste in amounts that vary depending on the type of system used for oil extraction. In fact, with the exclusion of the traditional discontinuous press system, which is now disused due to both economic and quality disadvantages, the largest amount of OMW is generated by the widely used three-phase centrifugal extraction technology that requires the addition of up to 50 L of water per 100 kg of olive paste [11]. Despite its variable composition–mainly due to factors such as olive variety, fruit ripeness, climatic conditions, and processing method–OMW contains high amounts of organic substances (from 4 to 18 g 100 g^−1^), including sugars, pectins, lignin, tannins, and lipids, a high content of phenolic compounds (0.5–24 mg L^−1^), as well as smaller amounts of inorganic substances (0.4–2.5 g 100 g^−1^), mainly potassium and phosphatic salts [12]. In particular, phenolic compounds, which are mainly responsible for the high polluting load and consequent phytotoxic activity of this discharging effluent, are also well known for their antioxidant properties, health-benefits and good bioavailability [13]. In particular, the most abundant bio-phenol found in OMW, as well as the most interesting from a nutritional point of view, is hydroxy-tyrosol, which exerts several biological activities, including free radical scavenging action, protection against the oxidation of human low-density lipoproteins (LDL), anti-inflammatory, antithrombotic, and in vitro anti-microbial activity [4,12,14,15]. Therefore, through the recovery and reutilization of such valuable compounds, the polluting waste from the olive oil industry could be turned into alternative and low-cost sources of bioactive phenols to be used as natural antioxidants in foods [16].

Besides improving nutritional value and providing health benefits, enrichment of food products with natural antioxidants might also be an effective tool to delay the lipid oxidation process and slow down the formation of off-flavors, thus extending the shelf life of the product [17,18,19,20]. In addition, natural antioxidants can be used as substitutes for other synthetic preservatives, such as butylated hydroxytoluene (BHT), butylated hydroxy anisole (BHA), *tert*-butyl hydroquinone (TBHQ), and propyl gallate (PG), that, despite their high efficiency in improving the shelf life of food products, have been shown to have harmful effects on human health [19,20].

Regularly eaten and affordable baked snacks like breadsticks–typical and widespread Italian bakery products appreciated in many other European countries–due to the way they intercept consumer and cultural food trends, could represent an ideal vehicle for the addition of natural bioactive compounds to the diet [21,22]. Breadsticks are crispy products with low moisture content and long shelf-life. The major causes of their quality deterioration are associated with loss of crunchiness and lipid oxidation [23]. In fact, despite being foods with low a_w_, breadsticks are usually prepared by adding high amounts of lipids–in some cases olive oil, but mostly vegetable oils and animal fat–to the basic formulation [22,24,25]. These phenomena are even more pronounced in the production of gluten-free (GF) baked products that, besides an unattractive appearance, poor mouthfeel and flavor, and a low nutritional value (lack of protein, iron, calcium, and vitamins), are more prone to staleness than their gluten-containing counterparts [26]. Such behavior may be due mainly to the higher amounts of water required to obtain workable doughs with acceptable consistency that, in the absence of gluten and in the presence of high amounts of starchy ingredients, foster moisture redistribution and starch retrogradation during storage, thus shortening the shelf life of the final product [27].

Recently, the authors of [25] reported the application of OL extracts as a useful tool to improve the oxidative stability and, in turn, the shelf-life of salty wheat-based baked snacks. However, after an extensive review of the available literature, no research on the potential use of olive oil by-products (neither OL nor OMW) in the development of both gluten containing and GF fortified baked snacks has been found.

In this context, the aim of this study is to evaluate the effects of the addition of two different amounts (500 and 1000 mg kg^−1^) of phenolic-rich extracts obtained from both OL and OMW on the textural and nutritional characteristics, as well as on the oxidative stability and consumer preferences, of GF breadsticks. Special emphasis will be placed on the assessment of both polyphenol fractions and antioxidant activity of the enriched baked products.

## 2. Materials and Methods

### 2.1. Raw Materials

Commercial rice flour, corn starch, guar gum and *Psyllium* fiber were from Chimab Campodarsego (Padova, PD, Italy). Fresh compressed yeast, salt and sugar were purchased from a local supermarket. Sunflower oil was from Carapelli Firenze (Florence, Italy).

### 2.2. Preparation of By-Product Extracts

#### 2.2.1. Olive Leaf Extract

The OL were collected in the crop season 2020/2021 from Coratina olive cultivar, stored at 4 °C, and processed in less than 24 h. After washing with tap water at room temperature, the olive leaves were dried at 120 °C for 8 min in a ventilated oven (Argolab, Carpi, Italy) to reach a moisture content <1%, and then milled with a blender (Waring-Commercial, Torrington, CT, USA). The extraction process was ultrasound-assisted (CEIA, Viciomaggio, Italy) and water was added in a ratio 1/20 (*w*/*v*). After three washings, each performed for 30 min at a temperature of 35 ± 5 °C, the extracts were filtered through Whatman filter paper (GE Healthcare, Milan, Italy), freeze-dried, and stored at −20 °C. Before the analysis, the extract was further filtered by using nylon filters (0.45 µm pore-size).

#### 2.2.2. Olive Mill Wastewater Extract

The OMW was collected in the crop season 2020/2021 from Ottobratica olive cultivar and produced according to a three-phase centrifugation process. The phenolic extract was obtained following the method previously reported by [28], with some modifications. Briefly, two liters of OMW were acidified to pH 2 with hydrochloric acid and washed three times with hexane (1:1, *v*/*v*) to remove the lipid fraction. The obtained mixture was vigorously shaken and centrifuged at 1550× *g* rpm for 3 min at 10 °C. The extraction procedure was carried out in separate funnels and repeated three times using ethyl acetate (1:4, *v*/*v*) as a solvent. The organic phase was then separated and filtered through a Buchner apparatus. The ethyl acetate was evaporated under vacuum in a rotary evaporator (Laborota400, Heidolph Instrument, Schwabach, Germany) at 25 °C. Finally, the obtained residue was dissolved in water to a final volume of 100 mL, further filtered by using a PTFE syringe filter (0.45 µm pore-size) and stored at 4 °C before analysis. To ensure that both hexane and ethyl acetate were removed (or were present in trace amounts) from the obtained extract, a headspace analysis using GC Thermo Trace 1310 apparatus (Waltham, MA, USA) equipped with a Single Quadrupole Mass Spectrometer ISQ LT system and a fused-silica capillary column (Thermo Scientific, Waltham, MA, USA) was carried out. It was found that the concentration of both the solvents used was below the limit of detection of the instrument.

#### 2.2.3. Total Polyphenol Content and Antioxidant Activity of OL and OMW Extracts

The total polyphenol content of both OL and OMW extracts (hereinafter referred to as OL_E_ and OMW_E_) was measured using the Folin-Ciocalteau reagent in a spectrophotometer (mod. Cary 3500, Agilent, Cernusco, Milan, Italy) set at 750 nm [29]. Briefly, after a proper dilution, an aliquot of 0.5 mL of extract were mixed with 0.5 mL of Folin-Ciocalteau reagent, 10 mL of sodium carbonate (7.5%) and adjusted to 25 mL with distilled water. The mixture was incubated in the dark for 1 h at room temperature before readings. Calibration curves were made using gallic acid and the results (mean of three replicates) were expressed as mg of gallic acid equivalents (GAE) per g of extract.

The free radical scavenging activity of both extracts was determined using a discoloration curve of the stable radical 2,2-diphenyl-1-picrylhydrazyl (DPPH). After proper dilution, an aliquot of 0.3 mL of extract was added to 2.7 mL of 0.0634 μmol mL^−1^ DPPH methanol solution for 1 h at 515 nm and 22 °C. Results (mean of three replicates) are expressed as decrease in absorbance (%) per mg of extract when 0.17 μmol of DPPH are available to react.

The determination of the phenolic compounds and antioxidant activity was carried out simultaneously in both the extracts and the breadstick samples only a few days after the extraction process (specifically, no more than 15 days later).

### 2.3. Preparation of GF Breadsticks

GF control breadsticks were prepared using a conventional GF formulation consisting of 50% rice flour and 50% corn starch as the basic recipe. The other ingredients used, which were added as % on flour basis, were: 55% water (26 °C), 10% sunflower oil, 4% compressed yeast, 3% sugar, 1.8% salt, 1.5% guar gum, and 1.5% *Psyllium* fiber. For the preparation of the enriched GF samples, OL_E_ and OMW_E_ were singly added to the basic formulation (on flour basis) a two different level of supplementation: low (500 mg kg^−1^) and high (1000 mg kg^−1^). Breadstick sample codes were defined according to the type of extract and the level of supplementation used, as follows: Control (no extract addition), Leaf50 (low addition of OL_E_), Leaf100 (high addition of OL_E_), WasteW50 (low addition of OMW_E_) and WasteW100 (high addition of OMW_E_). For each formulation, GF breadsticks were prepared by firstly suspending extracts, yeast, salt and sugar in aliquots of warm water. Then, these dissolved ingredients and the sunflower oil were slowly added to the pre-mixed dry ingredients and kneaded using a mixer (KitchenAid Professional, Model 5KSM7990, St. Joseph, MI, USA) equipped with a dough hook at speed 1 for 5 min followed by other 8 min at speed 2. After mixing, the obtained doughs (three for each sample) were proofed in a climate chamber for 30 min (33 °C–90% RH), manually shaped into 28 g weighted and 30 cm length sticks, placed on rectangular baked pans, and proofed once again for 30 min (33 °C–90% RH). Finally, the GF breadsticks were baked in an electric oven (Europa, Malo, VI, Italy) following a two-step baking process: they were firstly baked at 180 °C for 13 min, allowed to cool at room temperature for 30 min and then baked once again for other 22 min at 160 °C. After baking, breadstick samples were cooled for 1 h, before the analysis.

### 2.4. Breadsticks Measurements

#### 2.4.1. Moisture Content and Water Activity

The moisture content of the GF breadstick samples was determined using a moisture analyzer (Model Kern-DAB 100-3, KERN & SOHN GmbH, Balingen, Germany) equipped with a halogen quartz glass heater (400 W) and set with a standard heating profile at 105 °C. The results were expressed in % as the average of five repetitions.

Water activity was measured on ground and homogenized samples with an electronic hygrometer (model Aw-Win, Rotronic, Bassersdorf, Switzerland) equipped with a Karl-Fast probe previously calibrated in the range of 0.1–0.95 with solutions of lithium chloride (LiCl) of known activity. A total of five repetitions for each sample were made.

#### 2.4.2. Color Determination

Color measurements were carried out on the day of baking by using a tristimulus colorimeter (Minolta CR-300, Konica Minolta Sensing, Osaka, Japan) equipped with a measuring head CR-300 and previously calibrated against a white tile. To avoid inaccurate measurements due to the limited width of the samples, 60 breadsticks per batch were finely ground and placed into the granular material attachment (CR-A50, Konica Minolta Sensing, Osaka, Japan) of the colorimeter. The results were expressed in accordance with the Hunter Lab color space and the parameters acquired were lightness L*, redness (a*), and yellowness (b*). Total color difference (∆*E*) was also calculated by using the following equation:

∆*E* =((∆*L*^2^) + (∆*a*)^2^ + (∆*b*)^2^)^1/2^ (1)

For each sample, a total of ten repetitions were made.

#### 2.4.3. Textural Properties

Evaluation of breadsticks’ textural properties was carried out on twenty freshly prepared samples 1 h after baking by means of a three-point bending test. A texture analyzer (TA-XT2 Texture Analyzer, Stable Microsystems, Surrey, UK) equipped with a 30 kg load cell and a three-point bending rig (HDP/3PB) was used. After placing and blocking the two adjustable supports of the rig base at a span distance of 60 mm, a half breadstick–about 15 cm long–was placed centrally over the supports and broken by a blade probe moving downwards at a pre-test speed of 1 mm s^−1^ and a test speed of 3 mm s^−1^. The maximum peak force (N) required to break the sample and the distance to break (mm) were determined from the obtained force–distance curves and further referred to as hardness and brittleness. The software Texture Exponent TEE32 (v. 6.1.10.0 Stable Micro System, Surrey, UK) was used for data processing.

#### 2.4.4. Determination of Polyphenol Fractions and Antioxidant Activity

To determine the soluble, insoluble phenolic fractions of breadstick samples, the procedures previously described in GF breads by [26] were applied. Briefly, 2 g of finely ground breadstick samples were extracted twice with a solution (4 mL) of 37% hydrochloric acid/methanol/water (1/80/10, *v*/*v*/*v*). The supernatants were collected, filtered, and used for the determination of the soluble polyphenols. To obtain the insoluble phenolic fraction, sample residues from the soluble polyphenols’ extraction were digested with 5 mL of a methanol/concentrated sulfuric acid solution (10:1, *v*/*v*) for 20 h by shacking in a thermostatic water bath set at 85 °C. The obtained extracts were analyzed using the Folin-Ciocalteau reagent in a spectrophotometer (mod. Cary 3500, Agilent, Cernusco, Milan, Italy) set at 750 nm [29].

To estimate polyphenols’ bio-accessibility, the experimental samples were digested in vitro according to the procedure described by [26]. Briefly, 1 g of each ground sample was firstly digested with 0.5 mL of pepsin in a shaking water bath for 1 h at 37 °C to accurately simulate the gastric digestion, and then with 2.5 mL of a solution containing bile salts and the pancreatin enzyme and 2.5 mL of sodium chloride/potassium chloride solution (at room temperature for 2 h) to simulate the intestinal digestion. After removing protein by addition of trichloroacetic acid (20%, *v*/*v*), the digested extracts were spectrophotometrically analyzed as described for the soluble and insoluble polyphenols. For all the phenolic fractions, calibration curves were made using gallic acid as standard and the results (mean of three replicates) were expressed as mg of GAE 100 g^−1^ of breadsticks, d.m.

The free radical scavenging activity of the experimental GF samples was determined using the DPPH assay as previously described by [26], with some modifications. In brief, aliquots of 0.3 mL of organic extracts were made to react with 2.7 mL of 0.0634 μmol mL^−1^ DPPH methanol solution using a spectrophotometer (mod. Cary 3500, Agilent, Cernusco, Milan, Italy) set at 515 nm and 22 °C to obtain a decrease in absorbance by the radical DPPH. The absorbance was read at 1 min and every 5–10 min until the plateau was reached (70 min). The test was performed in triplicate and plots of μmol DPPH vs. time (min) were drawn. The antiradical activity (AR) was calculated using the following equation:

AR = ( (DPPH_initial_ − DPPH_plateau_) × 100 )/DPPH_initial_ (2)

#### 2.4.5. Determination of Oxidation Stability (Oxitest) and Shelf-Life Estimation

Oxitest (VELP Scientifica, Usmate Velate (MB), Italy) is an oxidation stability reactor that, according to AOCS International Standard Procedure Cd 12c-16 [30], allows rapid measurements of the stability of foods against the lipid oxidation by subjecting the sample to high temperature and pure oxygen overpressure. It is controlled through the specific OXISoft^TM^ Software, which also allows the prediction of oxidation stability for shelf-life studies. In the present research, 30 g of ground breadstick samples (10 g per plate in each oxidation chamber) were analyzed at different working temperatures (60, 70, 80, and 90 °C) and at a pressure of 6 bar. The induction period (IP) was calculated from the obtained pressure–time curves as the time required to achieve a 10% drop of the oxygen pressure inside the oxidation chambers. In case of linear dependence with the temperature, the software calculates a linear regression equation on a semi-log scale (log of the IP–temperature curve) to predict the estimated IP and, thus, the shelf-life (days) of the products at the desired storage temperature (25 °C). All measurements were made in duplicate.

#### 2.4.6. Sensory Evaluation

Sensory analysis was conducted to assess whether there were differences in preference for the freshly prepared breadsticks by performing two different quantitative affective tests. Firstly, a ranking preference test was carried out in two different sessions to allow separate comparisons between the control and the samples enriched with OL_E_ and OMW_E_, respectively. Subsequently, a paired comparison test was carried out to compare the two breadstick samples found to be the most preferred in the previous ranking tests, according to the preference degree.

The ranking test was performed in a laboratory and conducted in individual booths with a panel composed of 60 consumers (39 men and 21 women aged between 18 and 65 years). The samples were presented at room temperature in a randomized and balanced order and water was provided to allow appropriate cleansing of the palate between sample tastings. To establish whether there was a difference among samples for the given attribute (preference), data were analyzed using the Friedman’s test.

If the F_test_ value is higher than the F_critical_ value–which can be found in the *χ^2^* table for the defined confidence level (95%) and the degrees of freedom k-1–it can be argued that at least two samples are significantly different for the attribute analyzed. If it is established that a significant difference exists, to find out which samples differ from each other, the Fisher’s least significant difference (LSD) is calculated.

The paired comparison test was conducted using a panel composed of 76 consumers (42 men and 34 women aged between 18 and 65 years). In this case, data analysis was carried out by comparing the largest number of responses for one sample with the critical value reported in the statistical table for paired difference test (two tailed) that–at the defined confidence level (95%) and with the effective number of participants–indicates the minimum number of responses required to conclude that there is a significant difference between the two samples.

### 2.5. Statistical Analysis

Statistical analysis of the results was performed using Statistica 10.0 software (StatSoft, Inc., Tulsa, OK, USA). The experimental data were submitted to one-way analysis of variance (ANOVA) followed by Fisher’s least significant differences (LSD) to know the difference between each pair of means with 95% confidence. Pearson correlation analysis for relationships between some selected parameters was also used.

## 3. Results and Discussion

### 3.1. Total Phenolic Content and Antioxidant Activity of OL and OMW Extracts

As reported in Table 1, the total polyphenol content of OL and OMW samples was 134.7 ± 2.1 and 13.4 ± 0.2 mg GAE g^−1^, respectively, with the OL_E_ showing a phenolic concentration at about 10-fold higher than that found in OMW_E_. These results are considerable different from those previously reported in the literature for aqueous OL_E_, as well as for extracts obtained from OMW produced according to a three-phase centrifugation process [31,32]. However, considering that the amount of bioactive compounds accumulated in olive oil by-products may vary widely depending on many factors, the most important of which are pedoclimatic conditions, olive varieties, degree of maturity, and processing and extraction conditions, making an effective comparison of the data very difficult [33].

The antioxidant activity of the investigated extracts evidenced a similar trend, with the OL sample exhibiting a free radical scavenging capacity higher than that of the OMW_E_ (4.26 ± 0.08 vs. 0.32 ± 0.01).

Moreover, another important aspect that emerged from the comparison of the two experimental extracts and that deserves to be emphasized is related to the fact that OL rather than OMW seemed to be the best option, in terms of environmental resources, to obtain polyphenol-rich extracts. In fact, the aqueous extraction of phenolic compounds from OL, besides being the easiest, efficient, and solvent-free extraction procedure, allows for a higher yield with a low environmental impact.

### 3.2. Moisture Content and Water Activity of GF Breadisticks

As reported in Table 2, when comparing the control breadsticks to the fortified samples significant (*p* < 0.05) differences in terms of both moisture content and a_w_ were observed. All the enriched breadsticks showed moisture values (ranging from 9.24 to 11.50 g 100 g^−1^) significantly higher than the control (7.66 ± 0.07 g 100 g^−1^), the extent of the change being more pronounced at increasing level of supplementation for both the investigated extracts. In particular, the highest moisture values were observed in the sample Leaf100 (11.50 ± 0.12), closely followed by the sample Leaf50 (10.87 ± 0.14). Such an increase was probably due to the presence of fiber in the OL_E_, enabling the absorption of more water than the basic GF ingredients, thus suggesting a potential effect on the water absorption capacity of the resulting breadsticks.

A similar trend was observed for the a_w_, which significantly (*p* < 0.05) increased from a value of 0.41 observed in the control sample to a maximum value of 0.67 exhibited by the breadsticks prepared with the high level of addition of the OL_E_ (Table 2). As confirmation of this, values of correlation coefficients (r) revealed that higher moisture content corresponded to larger amount of available water (r = 0.985; *p* < 0.001). As expected, the data obtained in this study are considerably higher than those observed in wheat-based breadsticks by other authors, who reported moisture content ranging between 2.09 and 6.15 [23,34,35] and a_w_ values of about 0.14–0.38 [23,36]. Even though such differences should be not surprising given the higher amount of water usually required to obtain machinable GF dough with a proper consistency, it is worth noting that an increase in both moisture and a_w_, could lead to undesirable stability issues of the GF breadsticks during storage. In fact, in starch-based system, the presence of higher amounts of available or weakly associated water, which is not able to bind to starch as strongly as it does with protein, could lead to a faster moisture migration both within and out of the products, thus shortening their shelf life [37].

### 3.3. Color and Textural Properties of GF Breadisticks

Visual and textural characteristics play an important role in determining the final quality of baked products and, in turn, in influencing consumer choice. This is particularly true in GF baked products, which, unlike their gluten-containing counterparts, often exhibit an overly white coloration and a too hard, dry, and grainy texture that consumers find unappealing [27].

In terms of color features, all the enriched GF breadsticks exhibited the same lightness L* and yellowness (b* positive), but lower red (a* positive) values when compared to the control breadsticks, with no significant differences between samples prepared with the same supplementation level (Table 3). The only exception was observed in the sample WasteW100, which significantly differed from the control also in terms of b* values. In particular, all the fortified breadsticks showed a significant tendency (*p* < 0.05) to change from a red to a more greenish coloration, especially at the highest level of addition, probably as a consequence of the typical green and green to yellow color of the added OL_E_ and OMW_E_, respectively. However, as confirmed by the total color difference, which values were <1 for all the analyzed samples (Table 3), these observed differences were not obvious for the human eye, suggesting only a slight influence of both extracts on the color of the resulting breadsticks.

From a textural point of view, baked snacks like breadsticks are characterized by a rigid, stiff structure with a little tendency to deform before fracture when subjected to small forces [22]. In the present study, the different mechanical behavior of the experimental breadsticks was measured on the day of baking to assess their quality in terms of hardness and brittleness.

As reported in Table 3, both OL_E_ and OMW_E_ significantly (*p* < 0.05) lowered the maximum force needed to break the experimental breadsticks, irrespective of whether they have been added at low or high level. In particular, while the control sample showed the highest values of force at break (or the maximum resistance when broken) (51.57 ± 3.83 N), the most pronounced decrease in hardness values was observed in the samples enriched with the OL_E_ (at about 45 N for both samples). Since in low moisture food systems, water mainly acts as plasticizer [38], a possible explanation for this softening effect could be related to the higher moisture content and a_w_ observed in the samples enriched with OL_E_, closely followed by those enriched with the OMW_E_.

Brittleness, which is a textural parameter describing the distance traveled by the blade through the sample before its breaking and, thus, how far a sample can be deformed before fracture, did not show significant differences (*p* < 0.05) among the experimental samples. However, a slight increase of this parameter was observed in the sample Leaf50, suggesting a more leathery or rubbery behavior. The authors of [38] when measuring the mechanical properties equilibrated at different a_w_ of a particular baked snack, called dried bread, demonstrated that when values of a_w_ are higher than 0.56 the stress is released on rupture in an increasingly gradual manner, making prominent the ductile behavior and, thus, deformation over brittleness. However, it is noteworthy that the effect of hydration on the textural properties of baked cereal-based snacks is quite complex and varies depending on the basic formulation of the product, so that the critical values of both a_w_ and water content, corresponding to changes from a crispy to a more deformable behavior, may be different [39].

### 3.4. Polyphenol Fractions and Antioxidant Activity of GF Breadisticks

**Figure 1:**
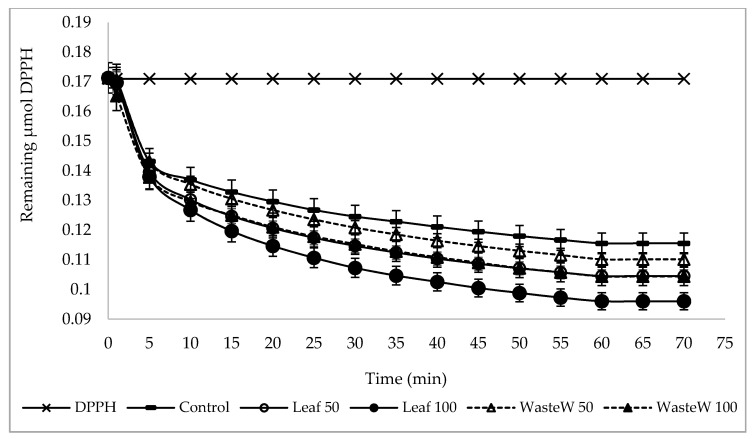
Time evolution of the DPPH curves in methanol of organic extracts from GF control and fortified breadsticks.

In the present study, the polyphenol fractions and the antioxidant activity of GF breadsticks enriched with natural phenolic-rich extracts from both OL and OMW were compared with a conventional unfortified GF breadstick sample. Results are summarized in Table 4 and Figure 1.

Among the experimental breadsticks, the control formulation exhibited amounts of total polyphenols significantly lower (162.87 ± 1.15 mg of GAE 100^−1^ d.m.) than those observed when the OL_E_ and OMW_E_ were individually added to the basic formulation (from 168.40 ± 1.86 to 189.28 ± 3.55 mg of GAE 100^−1^ d.m.) (Table 4). Only the sample WasteW50, which was prepared by adding the low percentages of OMW_E_, showed a total polyphenols content similar to that observed in the control, indicating that the most efficient increase was achieved by supplementing the basic recipe with the OL_E_ at both supplementation levels. In particular, the increment in the total phenolic content of the breadsticks enriched with low and high levels of OL_E_ was four-fold and two-fold higher than that observed by adding low and high levels of OMW_E_, respectively (14–16% vs. 3–8%)_._ These results were somewhat expected considering the higher polyphenol content observed in the OL_E_, which was at about 10-fold higher compared to that of the OMW_E_ (Table 1).

However, as it is well known, polyphenols can exist in the plant kingdom in both free and bound form. Therefore, to better evaluate the composition of the phenolic compounds in both control and fortified breadsticks, soluble and insoluble polyphenol fractions were also determined. As reported in Table 4, while the incorporation of both OL_E_ and OMW_E_ was significant (*p* < 0.05) in enhancing the soluble phenolic content of the resulting breadsticks, neither of the two affected the insoluble polyphenol fraction, which did not show significant differences among the investigated samples. In particular, the most significant changes were observed in the samples containing the OL_E_–with an increment with respect to the control ranging from 76% (Leaf50) to 126% (Leaf100)–followed by those prepared with the OMW_E_ (+32% and +56% for WasteW50 and WasteW100, respectively). Thus, the incorporation of the investigated phenolic-rich extracts led to a significant decrease (*p* < 0.05) in the insoluble/soluble polyphenols average ratio of the resulting breadsticks, an effect more prominent at increasing levels of addition, especially in the case of OL_E_ supplementation (Table 4). To better understand such an improvement, it must be born in mind that fruits and vegetables, compared with cereal grains like rice and corn–in which at about 70% of the total polyphenols exists in the bound forms–have most of their phenolic compounds in the free or soluble conjugate forms [40,41]. Therefore, the addition of extracts from plant wastes can favor the accumulation of the phenolic fraction more rapidly absorbed in the grastrointestinal tract, thus leading to an effective enrichment of the final products [42].

Since there is no direct relationship between the amount of polyphenols in foods and their bioavailability, to evaluate how many of the ingested phenolic compounds could be effectively absorbed and utilized by the human body, thus exerting their biological effects [43], the bio-accessible polyphenol fraction was also determined. As reported in Table 4, although all the fortified breadsticks showed amounts of bioaccessible polyphenols significantly higher than those observed in the control, the most significant increment in polyphenol bioavailability was achieved in the sample prepared with the highest level of OL_E_ (+23% with respect to the control), followed by the samples Leaf50 and WasteW100, wich behaved in a similar way (+14.5% and +15.1%, respectively). This enhancement effect was in line with the same effect previously described for the soluble polyphenol fraction, as also confirmed by the highly significant (*p* < 0.001) linear correlations observed between the soluble and bioaccessible fractions (r = 0.919). In fact, the contribution of the insoluble polyphenols to the bioavailability of the final product is usually lower than that of the soluble phenolic fraction, since they have to be released from the cell structure before being absorbed [41]. Interestingly, the authors of [44] demonstrated that the absorption and, consequently, the bioavailability of hydroxytyrosol in the gastrointestinal tract could be maximize after a supplementation of the diet with its naturally precursor oleuropein–which is the most abundant bio-phenol in the OL_E_–rather than with its free form (mainly present in the OMW_E_) or aglycone forms. However, to the best of our knowledge, data on soluble, insoluble and bioaccessible polyphenols in GF baked products are limited to only one previous study from the same authors, who found similar results in GF breads fortified with a natural apicultural product like bee pollen [26]. For this reason, a comprehensive comparison of the obtained data with the literature is difficult.

Very often, the main source of antioxidants in food products are represented by phenolic compounds, which are able to exert several biological functions, including antioxidant and free radical scavenging activity [4]. In the present study, the antioxidant activity of both control and enriched breadsticks was evaluated by using the DPPH radical scavenging assay, which is a method based on electron donation of antioxidants to neutralize the free radicals. As reported in Figure 1, the time evolution of the DPPH concentration curves in methanol of organic extracts from both control and GF breadsticks evidenced that all the fortified samples exhibited an antioxidant activity higher than the control. In particular, the OL_E_ seemed to be more effective in enhancing the scavenging activity against the stable radical DPPH of the resulting breadsticks, especially at the highest supplementation level (at about 44%). These findings are similar to those reported by other authors in wheat-based baked snacks enriched with OL_E_ [25]. As in the case of OL_E_, the OMW_E_, irrespective of whether it had been added at low or high level, also increased the antioxidant activity of the enriched samples, but the extent of this increase was significantly lower than that observed for the OL_E_, with the sample WasteW100 showing values similar to those observed in the sample Leaf50 (at about 38% and 39%, respectively) (Table 4). These results were in line with those previously observed for phenolic compounds. As a confirmation of this, significant positive correlations (*p* < 0.001) have been found between antiradical activity and soluble (r = 0.958), bioaccessible (r = 0.899), and total (r = 0.868) polyphenols.

### 3.5. Oxidation Stability (Oxitest) and Estimated Shelf-Life of GF Breadisticks

Lipid oxidation is one of the major causes of quality deterioration of dehydrated or low moisture bakery products–such as breadsticks and other bread substitutes–which usually require the addition of non-negligible amounts of fatty substances (from 5–15%, or even more, depending on their nature) to obtain desirable texture, appearance and flavor attributes [45]. Lipids, in fact, are susceptible to complex chemical changes that proceed through free-radical propagated chain reactions, which are triggered by unsaturated fatty acids reacting with oxygen. Other oxidation initiators, such as light or heat, certain enzymes, and metal ions are also involved in the process, by enhancing the lipid oxidation during storage [46]. The formation of free radicals and primary oxidation products, such as hydroperoxides, and their decomposition into secondary oxidation products, such as aldheydes, ketones, and hydrocarbons, are directly responsible for the formation of undesirable flavors in rancid foods. Therefore, the use of natural antioxidants–such as OL_E_ and OMW_E_–well known for their ability to protect against free radicals, may be an effective way to promote the oxidation stability of functional GF breadsticks, thus exending their shelf-life [23]. In the present study, the effect of the investigated extracts on the oxidation stability of both GF control and fortified breadsticks was evaluated by using accelerated oxidation tests performed in the OXITEST reactor at four different working temperatures (60, 70, 80, and 90 °C) and at a costant oxygen overpressure (6 bar) to allow the estimation, in a short period of time, of the potential shelf-life of the experimental samples. Monitoring the drop in the oxygen pressure inside the oxidation chambers–which correspond to a certain level of detectable rancidity or a rapid change in the oxidation rate–evidenced that, while the control samples exhibited the lowest lipid stability to oxidation (at about 3 and 52 h at 90 and 60 °C, respectively), the addition of both extracts significantly increased the IP of the fortified breadsticks at all the working temperatures, with the samples WasteW100 being more stable (at about 5 and 99 h at 90 and 60 °C, respectively) than the samples WasteW50 (3 and 57 h at 90 and 60 °C), Leaf100 (3 and 60 h at 90 and 60 °C), and Leaf50 (2 and 58 h at 90 and 60 °C), respectively. Starting from this point and considering that with increasing working temperature the IP of the oxidative reaction decreased, thus suggesting a linear dependence of oxidation stability and temperature, the potential shelf-life of the experimental samples at the storage temperature of 25 °C was estimated by using linear regression equations on a semi-log scale. The estimated shelf-life of the GF breadsticks based on lipid oxidation data is shown in Table 5.

As can be seen, the incorporation of increasing percentages of both OL_E_ and OMW_E_ was followed by a concurrent increase in the estimated shelf-life of the resulting breadsticks (Table 5), indicating an effective role of antioxidants in preserving the final products. In particular, the greatest values were observed in the sample prepared with the high level of addition of OMW_E_ which nearly doubled the shelf-life of the control, closely followed by the sample WasteW50 (+48% increment with respect to the control). A significant shelf-life extension was also registered in those breadsticks prepared with the addition of OL_E_, but the extent of this increase was significantly lower than that observed for the OMW_E_ extracts at both supplementation levels (+23 and 32% for Leaf50 and Leaf100, respectively). These findings, however, were somewhat unexpected considering the opposite results observed in terms of antioxidant activity among the fortified samples. In fact, in spite of a lower (or similar) antioxidant activity (Table 4), the OMW_E_-enriched breadsticks seemed to be kept fresh for longer, at least in terms of oxidation stability, than those prepared with the OL_E_, irrespective of the level of substitution used. A possible explanation of this contrasting behavior may be related to the significant differences recorded in the a_w_ values among OL_E_ and OMW_E_ breadsticks (Table 2). In fact, it has been demonstrated that water can play both pro-oxidant and antioxidant roles in lipid oxidation, depending on whether its content in the food is within or above the monolayer moisture content [47,48]. At low levels of a_w_–near to the monolayer range–water can exhibit an antioxidant role by forming a barrier that protects the sensitive sites from reactions with oxigen, but also by lowering metal catalytic activity, increasing hydration of hydroperoxides (and consequently decreasing the rate of free radicals formation), as well as by promoting recombination of free radicals. In contrast, at higher a_w_ values, as is the case with the experimental OL_E_-enriched breadsticks, water can show a pro-oxidant role by acting as a plasticizing agent, thus promoting mobility and solubilization of catalysts, as well as by inducing matrix swelling, thus exposing new reactive sites [47,48]. Therefore, the lower oxidative stability observed in the breadsticks fortified with the OL_E_ in comparison to that observed in those enriched with the OMW_E_ might suggest that, in such a complex matrix. the effect of a_w_ is greater than the conservative role exerted by the added antioxidants.

However, considering that in low moisture foods the causes of lipid oxidation are still not completely understood and that both monolayer and glass transition theories have led to contrasting results when used to predict lipid oxidation rates as a function of a_w_ [48], further studies are needed to give consistency to the obtained results.

### 3.6. Sensory Evaluation of GF Breadisticks

In the first two sessions of the sensory evaluation, participants were asked to rank the overall preferences for the freshly prepared breadsticks, by comparing the control to the samples enriched with both OL_E_ and OMW_E_ separately. As reported in Table 6, the obtained results evidenced that no sample was significantly preferred to another in both comparisons.

More specifically, when comparing the control sample to the OL_E_-enriched breadsticks, the lowest ranking score was assigned to the sample Leaf100, followed by the Leaf50 and the control breadsticks (Table 6(a)), suggesting a clear tendency for consumers to recognize the fortified samples as the most palatable choice. However, as evidenced by the obtained F_test_ value (4.03), which was lower than the F_critical_ value reported in in the χ^2^ table (5.99), the recorded difference in the preference degree was not significant. A similar trend was also observed when comparing the control sample to the two breadsticks fortified with OMW*_E_* (Table 6(b)). In this case, although the differences in the preference degree assigned to the three samples were less pronounced (F_test_ value: 1.13 and F_critical_ value: 5.99), a consumer’s tendency to prefer the breadsticks prepared with the high level of OMW_E_ could also be observed.

**Figure 2:**
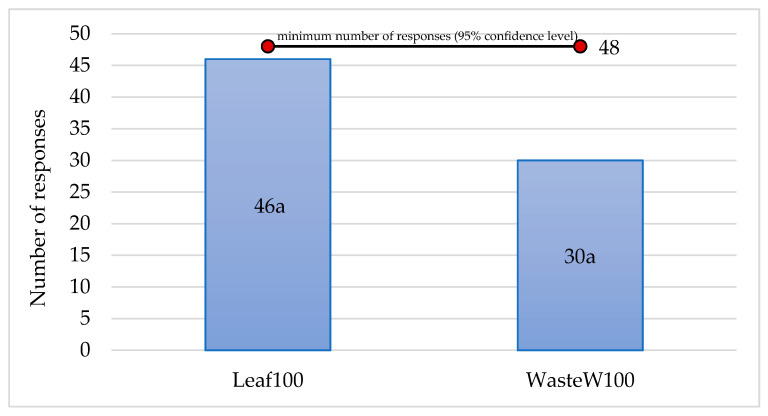
Differences in the number of responses accorded by consumer to the Leaf100 and WasteW100 samples in the paired comparison test.

Based on these results, Leaf100 and WasteW100 samples were then subjected to a paired comparison test to assess if a significant difference exists between them in terms of preference (Figure 2).

According to the statistical table for paired difference test (two tailed), 48 is the minimum number of responses needed to conclude that a preference exists between two samples at the selected significance level (5%) and with a total number of assessors of 76. This value was not reached by either of the two experimental breadsticks, even if the sample Leaf100 came very close to this minimum number (Figure 2). However, it should be noted that, although the number of responses did not differ significantly between the two enriched breadsticks, the sample Leaf100 was preferred by the 61% of the assessors, indicating a marked, but not significant, tendency for consumers to consider it as the most appetizing choice.

## 4. Conclusions

Data obtained in the present study evidenced, for the first time, that the incorporation of phenolic-rich extracts from olive oil by-products in GF formulations can be considered a successful strategy in the preparation of technologically viable functional breadsticks with extended shelf-life. Although all fortified samples, especially those enriched with the OL_E_, were softer, they also exhibited a similar crumbly texture and minimal color changes compared to the control, indicating an only small impairment in their technological feasibility. This was also confirmed by the sensory evaluation, which showed a marked (but not significant) tendency of consumers to consider the enriched breadsticks, especially those prepared with the high percentage of OL_E_, as the most preferred. The incorporation of both extracts also resulted in improved nutritional and functional properties of the final breadsticks, as evidenced by the changes observed in the insoluble/soluble polyphenol ratio in favor of the soluble fraction, by the enhanced bioavailability of polyphenols, as well as by the higher antioxidant activity, especially in those samples prepared with the higher percentage of OL_E_. Furthermore, all the fortified breadsticks exhibited higher stability against the lipid oxidation and, in turn, an extended estimated shelf-life, even if, in this case, better behavior was observed in the samples prepared with both levels of OMW_E_.

In conclusion, the best results were achieved in those samples fortified with the high percentage of OL_E_ in which, in spite of a slightly shorter shelf-life, a more proper balance among the technological, sensory and functional properties was observed. Further experiments are needed to give consistency to these preliminary findings, especially to better clarify the influence of both antioxidant activity and a_w_ in the estimation of the shelf-life of GF low moisture baked snacks.

## Figures and Tables

**Table 1:** Total polyphenol content and antioxidant activity of olive leaf and oil mill wastewater extracts.

Samples ^1^	Total Polyphenols (mg GAE g^−1^, As Is)	Antioxidant Activity (% per mg of Extract)
OL_E_	134.7 ± 2.1 a	4.26 ± 0.08 a
OMW_E_	13.4 ± 0.2 b	0.32 ± 0.01 b

^1^ Mean value ± standard deviation. Within columns, values (mean of three repetitions) with the same letter do not differ significantly from each other according to Least Significant Difference (LSD) test (*p* < 0.05). OL_E_: olive leaf extract; OMW_E_: olive mill wastewater extract. GAE: gallic acid equivalent.

**Table 2:** Moisture content and a_w_ of gluten-free (GF) breadsticks.

Characteristics	Samples ^1^				
	Control	Leaf50	Leaf100	WasteW50	WasteW100
Moisture (g 100 g^−1^)	7.66 ± 0.07 e	10.87 ± 0.14 b	11.50 ± 0.12 a	9.24 ± 0.08 d	10.25 ± 0.09 c
a_w_	0.406 ± 0.00 e	0.655 ± 0.00 b	0.667 ± 0.01 a	0.547 ± 0.00 d	0.593 ± 0.00 c

^1^ Mean values ± standard deviation. Within rows, values (mean of five replicates) with the same letter do not differ significantly from each other according to LSD test (*p* < 0.05).

**Table 3:** Textural and color properties of GF breadsticks.

Characteristics	Samples ^1^				
	Control	Leaf50	Leaf100	WasteW50	WasteW100
Color properties					
*L*	60.10 ± 1.16 a	59.85 ± 0.82 a	60.55 ± 0.4 a	60.88 ± 1.09 a	60.67 ± 0.65 a
*a*	1.59 ± 0.05 a	1.40 ± 0.08 b	1.25 ± 0.04 c	1.46 ± 0.08 b	1.24 ± 0.08 c
*b*	12.93 ± 0.10 a	12.63 ± 0.37 ab	12.69 ± 0.31 ab	12.93 ± 0.17 a	12.39 ± 0.21 b
Δ*E*	–	0.13	0.22	0.08	0.23
Textural properties					
Hardness (N)	51.57 ± 3.83 a	45.34 ± 5.02 c	45.07 ± 3.24 c	49.12 ± 3.05 b	48.39 ± 2.74 b
Brittleness (mm)	0.70 ± 0.08 a	0.80 ± 0.15 a	0.72 ± 0.18 a	0.65 ± 0.11 a	0.69 ± 0.14 a

^1^ Mean values ± standard deviation. Within rows, values (means of 10 repetitions for color measurements and 20 repetitions for textural properties) with the same letter do not differ significantly from each other according to LSD test (*p* < 0.05).

**Table 4:** Polyphenol fractions and antioxidant activity of GF control and fortified breadsticks.

Characteristics	Samples ^1^				
	Control	Leaf50	Leaf100	WasteW50	WasteW100
Polyphenol fractions (mg GAE/100 g d.m.)					
Soluble	20.32 ± 0.97 e	35.85 ± 1.36 b	46.26 ± 2.47 a	27.05 ± 1.05 d	31.81 ± 0.90 c
Insoluble	142.4 5± 1.64 a	149.76 ± 5.56 a	143.02 ± 1.51 a	141.35 ± 1.93 a	143.71 ± 3.56 a
IP/SP	6.98 a	4.18 c	3.10 d	5.23 b	4.52 c
Total ^2^	162.87 ± 1.15 c	185.61 ± 6.56 a	189.28 ±3.55 a	168.40 ± 1.86 bc	175.52 ± 4.43 b
Bio-accessible	113.51 ± 2.30 d	129.93 ± 2.44 bc	139.68 ± 2.46 a	125.29 ± 1.09 c	130.64 ± 4.35 b
Δ bio-accessibility (%)	-	14.5	23.0	10.4	15.1
Antioxidant activity ^3^ (%)	32.28 ± 2.19 d	38.74 ± 0.24 b	43.84 ± 0.93 a	35.69 ± 0.17 c	37.59 ± 1.08 bc

^1^ Mean values ± standard deviation. Within rows, values (mean of three repetitions) with the same letter do not differ significantly from each other according to LSD test (*p* < 0.05). ^2^ The total polyphenol content was calculated as the sum of the soluble and insoluble fractions. ^3^ Corresponding to 36 mg of breadsticks, which consumed these percentages when 0.17 μmol of 2,2-diphenyl-1-picrylhydrazyl (DPPH) are available for reaction. GAE: gallic acid equivalent; IP/PS: insoluble polyphenols/soluble polyphenols ratio.

**Table 5:** Estimated shelf-life of GF breadsticks based on lipid oxidation data (day at 25 °C).

Characteristics	Samples ^1^				
	Control	Leaf50	Leaf100	WasteW50	WasteW100
Estimated shelf-life	62 ± 2 e	76 ± 3 d	82 ± 2 c	92 ± 6 b	116 ± 1 a
*R* ^2^	0.999	0.999	0.999	0.999	0.997

^1^ Mean values ± standard deviation. Within rows, values (mean of two repetitions) with the same letter do not differ significantly from each other according to LSD test (*p* < 0.05).

**Table 6:** Results of the ranking preference tests on the freshly prepared GF breadsticks.

Samples	(a) ^1^			(b)		
	Control	Leaf50	Leaf100	Control	WasteW50	WasteW100
Rank sum	131	120	109	125	122	113
Significance *p* < 0.05	a	a	a	a	a	a

^1^ (a) comparisons among the control sample and the OL_E_-enriched breadsticks; (b) comparisons among the control sample and the OMW_E_-enriched breadsticks.

## Data Availability

Not applicable.
